# Correction to: Upregulating MicroRNA-410 or Downregulating Wnt-11 Increases Osteoblasts and Reduces Osteoclasts to Alleviate Osteonecrosis of the Femoral Head

**DOI:** 10.1186/s11671-020-03465-z

**Published:** 2021-03-09

**Authors:** Yukun Yin, Lixiang Ding, Yu Hou, Haoran Jiang, Ji Zhang, Zhong Dai, Genai Zhang

**Affiliations:** 1 Department of Traditional Chinese Medicine, National Cancer Center/National Clinical Research Center for Cancer/Cancer Hospital, Chinese Academy of Medical Sciences and Peking Union Medical College, Beijing, 100021 China; 2 Department of Spine, Beijing Shijitan Hospital, Capital Medical University, No. 10 Tieyi Road, Yangfangdian, Haidian District, Beijing, 100038 People’s Republic of China; 3 Department of General Medicine, Huanxing Cancer Hospital, Chaoyang District, Beijing, 100005 People’s Republic of China

## Correction to: Nanoscale Res Lett (2019) 14:383 10.1186/s11671-019-3221-6

After publication of the original article [1], the authors flagged that their article had published with an incomplete version of affiliation ‘1’.

The original article has since been corrected with the complete version of the affiliation, and the complete affiliation can be found in this correction.
